# Editorial: Advances in protein structure biology – use of AI and beyond

**DOI:** 10.3389/fmolb.2026.1903318

**Published:** 2026-06-25

**Authors:** Arumay Pal, Emmanouela Filippidi, Sucharita Dey

**Affiliations:** 1 Department of Pharmaceutical Sciences, Retzky College of Pharmacy, University of Illinois Chicago, Chicago, IL, United States; 2 Department of Materials Science and Engineering, University of Crete, Heraklion, Greece; 3 IESL-FORTH, Heraklion, Greece; 4 Max Planck Institute of Molecular Cell Biology and Genetics, Dresden, Germany; 5 Department of Bioscience and Bioengineering, Indian Institute of Technology, Jodhpur, Rajasthan, India

**Keywords:** artificial intelligence (AI), disease mutations, protein engineering, protein-protein interaction, structural biology, structure prediction, therapeutic discovery

## Introduction

**FIGURE 1:**
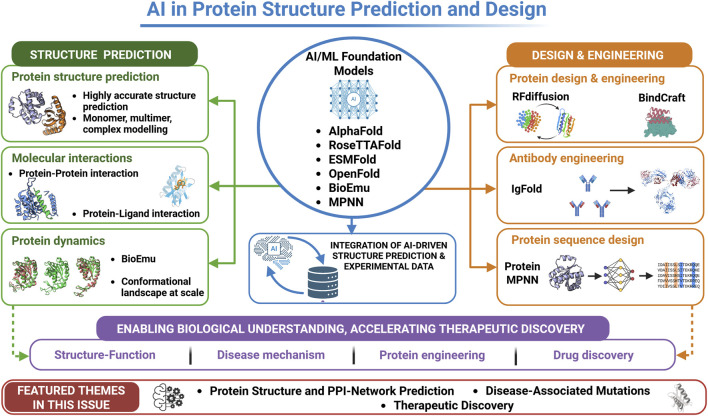
A comprehensive look on the applications of recently developed AI models in the field of protein structure prediction and design. The bottom panel shows the thematic areas of the featured articles in the present research topic. Figure is made using licensed version of BioRender (https://www.biorender.com/).

Artificial intelligence (AI) has driven major technological breakthroughs in structural biology over the past several years, with the advent of AlphaFold and RoseTTAFold in 2020 and the subsequent advance to AlphaFold 3 in 2024 (Jumper et al., 2021; Abramson et al., 2024; Humphreys et al., 2021). Together with structure prediction models such as ESMFold (Lin et al., 2023) and ensemble-sampling frameworks like BioEmu (Lewis et al., 2025), these approaches now form a foundational computational infrastructure underpinning applications ranging from protein engineering to drug discovery (Figure 1).

Perhaps the most transformative impact of AI/ML in structural biology is being realized in protein design and engineering. Deep learning–based generative models are revolutionizing *de novo* protein design, enabling the creation of proteins, enzymes, binders, and therapeutic candidates with predefined structures and functions. Frameworks such as Rfdiffusion and BindCraft have demonstrated remarkable success in designing high-affinity protein binders and novel protein architectures, with these advances increasingly validated by experimental studies (Watson et al., 2023; Pacesa et al., 2025). Complementary approaches, including ProteinMPNN for sequence design and IgFold for antibody structure prediction, have further expanded the ability to engineer functional proteins directly from structural or sequence information (Dauparas et al., 2022; Ruffolo et al., 2023). Open-source initiatives such as OpenFold are democratizing access to state-of-the-art protein AI models, enabling academic and industrial researchers to adapt and train models on proprietary datasets while maintaining data privacy (Ahdritz et al., 2024). Beyond structure prediction and protein design, emerging AI methodologies are enabling the characterization of biomolecular dynamics, conformational landscapes, molecular recognition, and protein-protein and protein-ligand interactions at unprecedented scale and resolution, providing deeper mechanistic insights into biological processes and thereby accelerating therapeutic discovery (Lewis et al., 2025; Liao et al., 2025).

This Research Topic, “Advances in Protein Structure Biology–Use of AI and Beyond”, brings together six contributions-three original research articles, two reviews, and one methods paper-that collectively showcase the expanding role of AI in structural biology, from protein structure prediction and interactome analysis to disease mechanisms and therapeutic discovery.

## Overview of featured articles

A central theme of this issue highlights how AI is transforming the prediction of protein structures and protein-protein interaction (PPI) networks. In their mini-review, Yin et al. detail the evolution of AI-driven structure prediction, tracing its trajectory from growing impact in biological research to practical breakthroughs such as enzyme engineering and pathogenic misfolding. To address PPI network prediction, Wang et al. introduce a hypergraph-based framework that leverages hierarchical network compression to improve protein complex identification in dense datasets. Specific to plant systems, Dickey et al. review recent advances in experimental and computational approaches to mapping PPIs, highlighting both the opportunities and challenges of integrating AI-based predictions with experimental interactome data to achieve more comprehensive network coverage.

A second theme centers on the molecular impacts of disease-associated mutations. Saito et al. combined structural modeling with clinical genomics to investigate an unexplained missense mutation in the DNA2 helicase. By mapping the altered ATP-binding geometry, they showed how the mutation locks the protein into an inactive conformation, shedding light on the molecular causes of unresolved genetic disorders. From a methodological standpoint, Leyva et al. introduced a scalable workflow using fragment-based density functional theory (DFT) to profile TP53 variants. By combining whole-exome sequencing, protein structure prediction, and quantum-chemical calculations, they successfully linked specific electronic signatures to protein dysfunction, offering a promising approach to interpreting cancer-associated mutations.

A third theme illustrates the growing convergence of AI and therapeutic discovery. Pandi et al. demonstrate the synergy between experimental assays and AI-powered molecular modeling to identify bioactive compounds from *Adansonia digitata*. By combining deep learning-based docking with molecular dynamics simulations, the research provides mechanistic insights into the plant’s anticancer and antibiofilm properties.

Collectively, these contributions highlight the increasingly diverse roles of AI in structural biology—from predicting protein structures and interaction networks, to interpreting disease-associated mutations, and accelerating the discovery of new therapeutic strategies. Together, they underscore a broader shift toward AI-driven integrative computational-experimental workflows that are reshaping the study of biological structure, function, and disease.
